# Supplementary material for: Multi-trait analysis of rare-variant association summary statistics using MTAR
Source: Nat Commun. 2020 Jun 5;11:2850. doi: 10.1038/s41467-020-16591-0 (PMC7275056; doi:10.1038/s41467-020-16591-0)
Supplement: Supplementary file 1 — Supplementary Information [file 41467_2020_16591_MOESM1_ESM.pdf]

Multi-trait analysis of rare-variant association summary statistics using MTAR

Luo et al.

Supplementary Information for "Multi-trait analysis of rare-variant association summary statistics using MTAR"

Lan Luo,[1,†] Judong Shen,[2,†] Hong Zhang,[2] Aparna Chhibber,[3] Devan V. Mehrotra,[4] and Zheng-Zheng Tang[5,6,*]

[1] Department of Statistics, University of Wisconsin-Madison, Madison, WI 53706, USA

[2] Biostatistics and Research Decision Sciences, Merck & Co., Inc., Rahway, NJ 07065 USA

[3] Genetics and Pharmacogenomics, Merck & Co., Inc., West Point, PA 19446 USA

[4] Biostatistics and Research Decision Sciences, Merck & Co., Inc., North Wales, PA 19454 USA

[5] Department of Biostatistics and Medical Informatics, University of Wisconsin-Madison, Madison, WI 53715, USA

[6] Wisconsin Institute for Discovery, Madison, WI 53715, USA

† These authors contributed equally to this work.

∗ Correspondence to Zheng-Zheng Tang: tang@biostat.wisc.edu

Supplementary Methods

Non-polymorphic sites. For a gene with m rare variants, if for trait k, there are only m' ($m' < m$) polymorphic SNPs, then the covariate matrix of genetic effects $\mathbf{B}$, the estimators $\widehat{\boldsymbol{\beta}}$ and their covariance matrix $\boldsymbol{\Sigma}$ need to be updated accordingly. Assume in a simple case, there are 3 SNPs in 3 traits. Trait 1 only has 1 SNP that is polymorphic. Let $\mathbf{B}_2 = \{a_{ij}\}_{i,j=1}^{K}$, $\mathbf{B}_1 = \{c_{ij}\}_{i,j=1}^{m}$ and the LD correlation matrix $\mathbf{R} = \{r_{ij}\}_{i,j=1}^{m}$, then the covariance matrix $\mathbf{B}$ has the form

$$\mathbf{B}_{7\times 7} = \begin{pmatrix} a_{11}c_{11} & a_{12}(c_{11}\ c_{12}\ c_{13}) & a_{13}(c_{11}\ c_{12}\ c_{13}) \\ a_{21}\begin{pmatrix} c_{11} \\ c_{21} \\ c_{31} \end{pmatrix} & a_{22}\begin{pmatrix} c_{11} & c_{12} & c_{13} \\ c_{21} & c_{22} & c_{23} \\ c_{31} & c_{32} & c_{33} \end{pmatrix} & a_{23}\begin{pmatrix} c_{11} & c_{12} & c_{13} \\ c_{21} & c_{22} & c_{23} \\ c_{31} & c_{32} & c_{33} \end{pmatrix} \\ a_{31}\begin{pmatrix} c_{11} \\ c_{21} \\ c_{31} \end{pmatrix} & a_{32}\begin{pmatrix} c_{11} & c_{12} & c_{13} \\ c_{21} & c_{22} & c_{23} \\ c_{31} & c_{32} & c_{33} \end{pmatrix} & a_{33}\begin{pmatrix} c_{11} & c_{12} & c_{13} \\ c_{21} & c_{22} & c_{23} \\ c_{31} & c_{32} & c_{33} \end{pmatrix} \end{pmatrix}.$$

The estimate becomes $\widehat{\boldsymbol{\beta}} = (\widehat{\beta}_1, \widehat{\boldsymbol{\beta}}_2^T, \widehat{\boldsymbol{\beta}}_3^T)^T = (\widehat{\beta}_{11}, \widehat{\beta}_{21}, \widehat{\beta}_{22}, \widehat{\beta}_{23}, \widehat{\beta}_{31}, \widehat{\beta}_{32}, \widehat{\beta}_{33})^T$ and their covariance matrix $\boldsymbol{\Sigma}$ will have reduced dimension accordingly.

Supplementary Figures

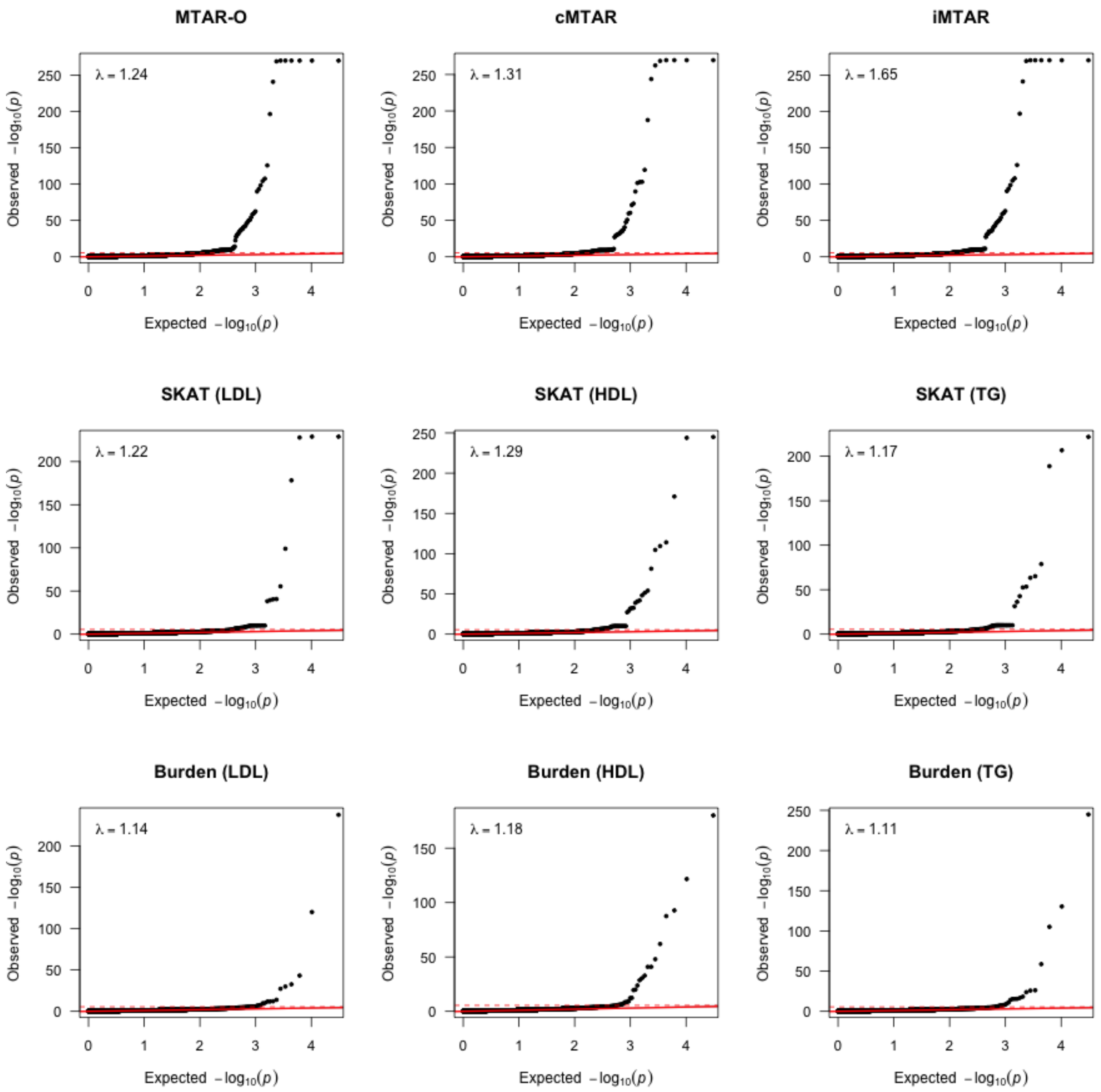

Supplementary Figure 1 QQ plots of MTAR-O, cMTAR, iMTAR, and single-trait SKAT and burden test results in the GLGC data analysis.

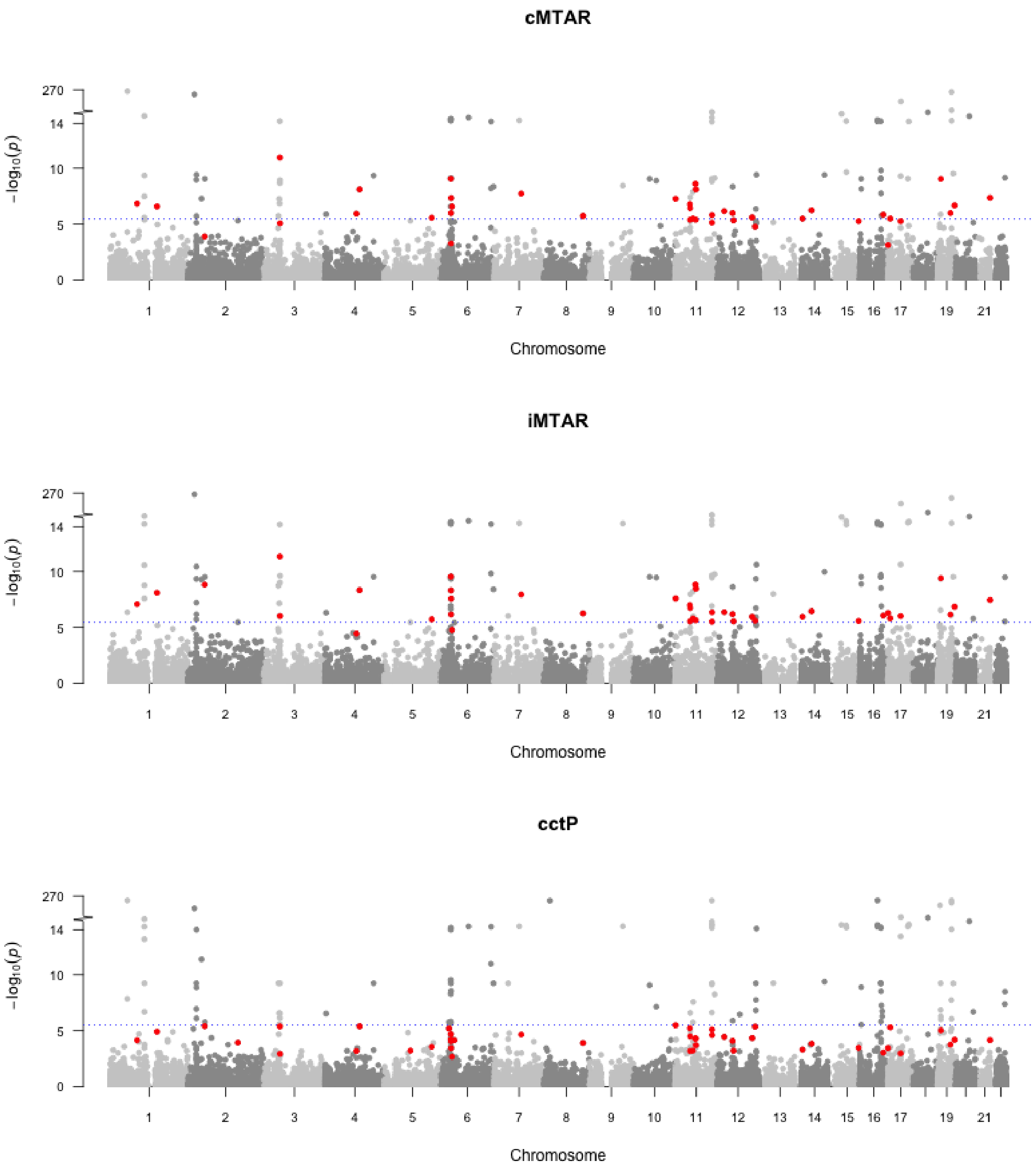

Supplementary Figure 2 Manhattan plots of cMTAR, iMTAR and cctP results in the GLGC data analysis. The horizontal line marks the genome-wide significance threshold (3.3×10^{-6}). The 41 genes highlighted in red are those exclusively discovered by MTAR tests (MTAR-O, cMTAR, and iMTAR). The Manhattan plots for the MTAR-O and minP are shown in Fig. 3.

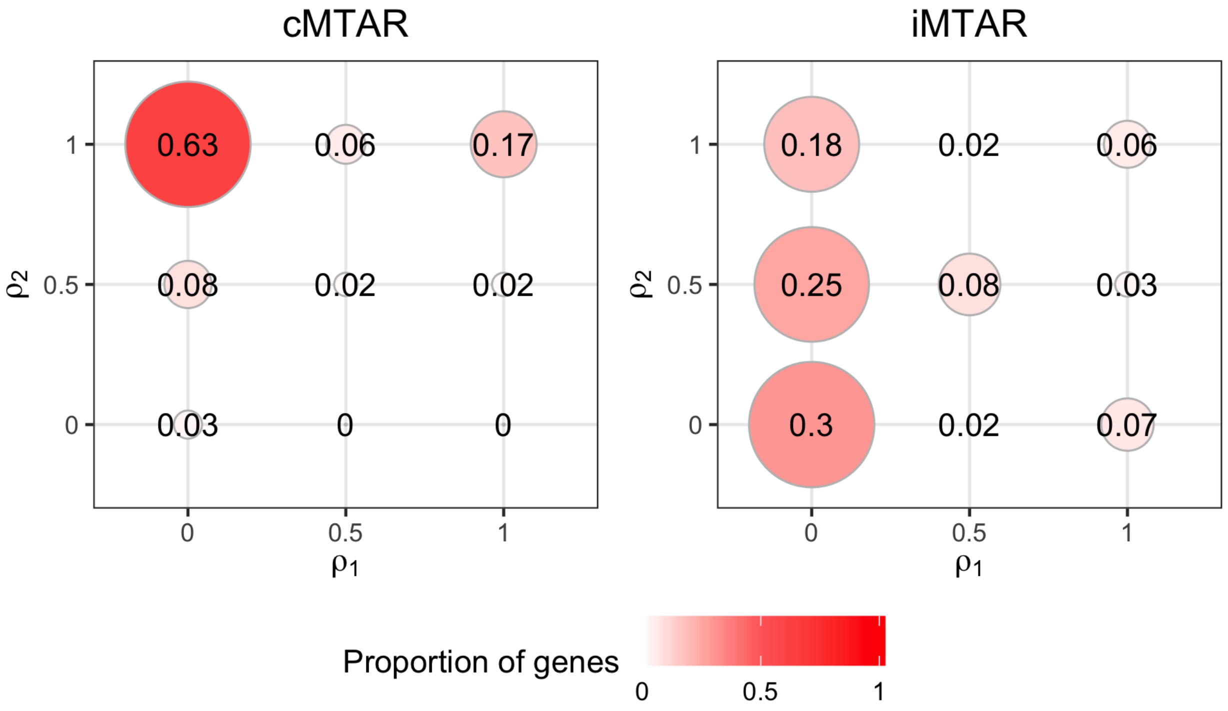

Supplementary Figure 3 Distribution of optimal values of ρ_1 and ρ_2 among the 107 and 131 genes repectively identified by cMTAR and iMTAR in the GLGC data analysis.

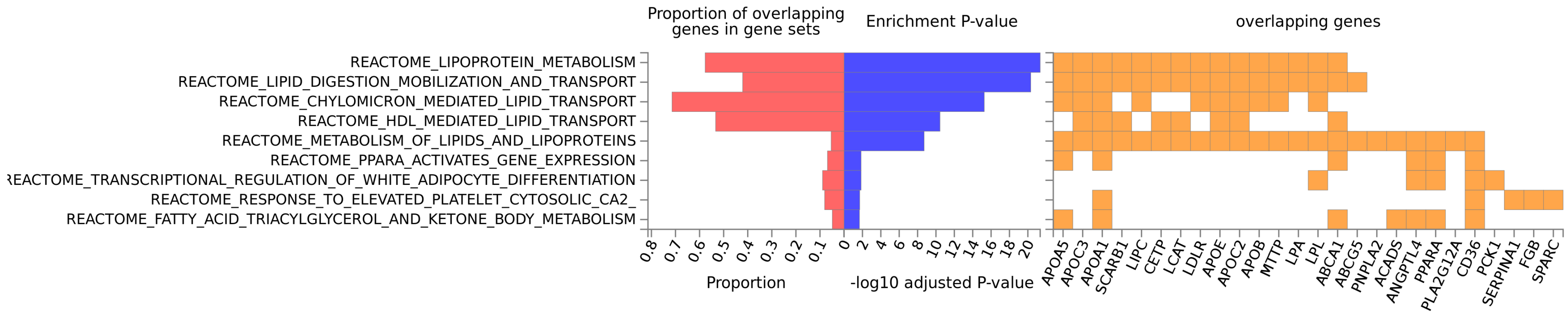

Supplementary Figure 4 Gene set enrichment results for the 139 MTAR identified genes using the one-sided hypergeometric test against Reactome Pathways.

a

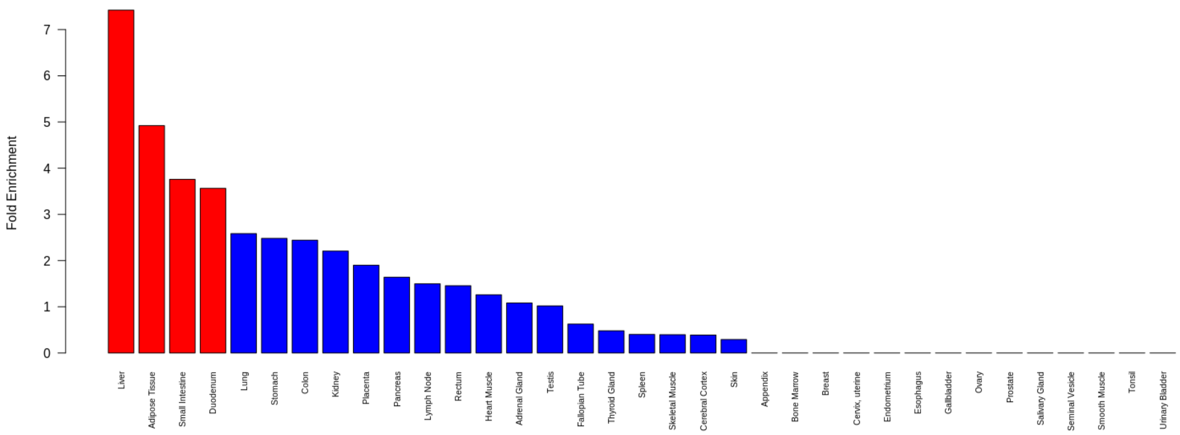

b

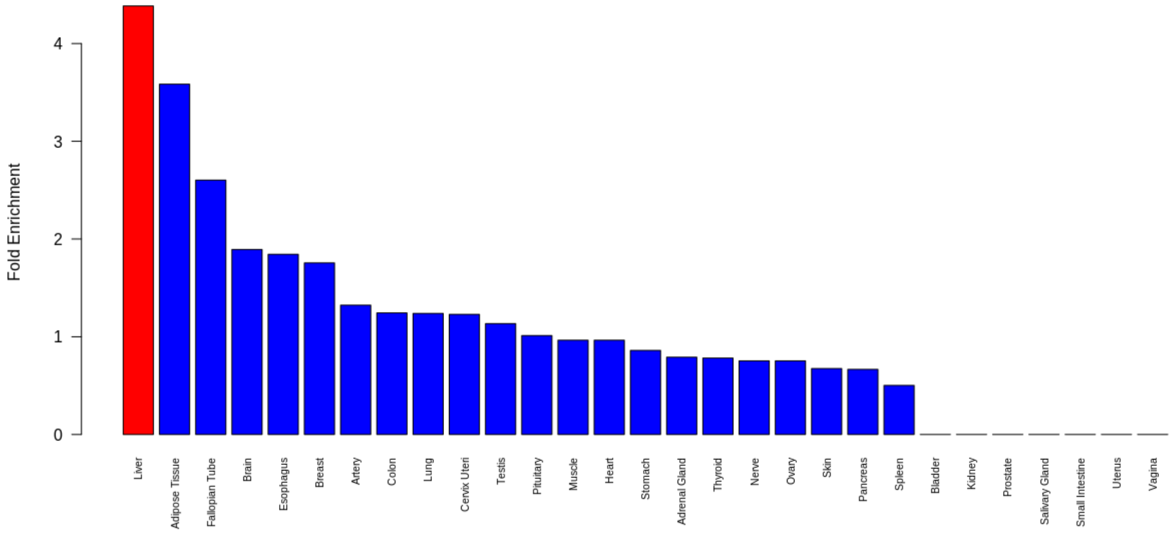

Supplementary Figure 5 Tissue enrichment analysis. **a.** All 139 significant genes using tissue specificity defined from Human Protein Atlas RNA-seq data; **b.** All 139 significant genes using tissue specificity defined from GTEx RNA-seq data. Tissues in red had false discovery rate adjusted enrichment P-values from the one-sided hypergeometric test < 0.05. Specifically, the P-values for liver, adipose tissue, smaller intestine, and duodenum are 8.0×10^{-15}, 1.2×10^{-2}, 6.0×10^{-3}, and 1.1×10^{-2} in **a**; the P-value for liver is 1.3×10^{-3} in **b**.

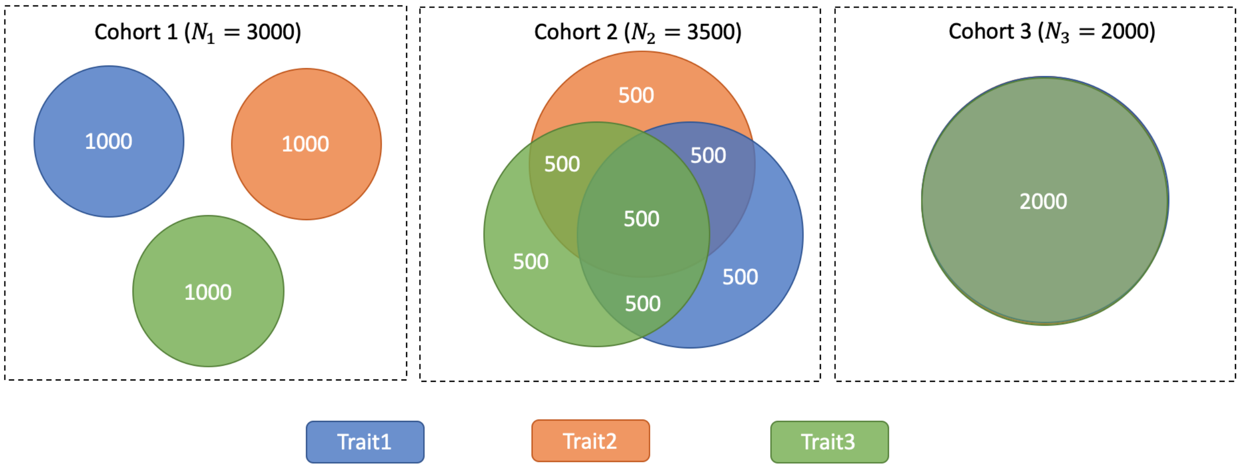

Supplementary Figure 6 Venn diagram on the samples among three traits in the three simulated cohorts. In cohort 1, each subject has one trait measured; in cohort 2, part of the subjects have all three traits measured and part of the samples has one or two traits measured; in cohort 3, each subject has all three traits measured.

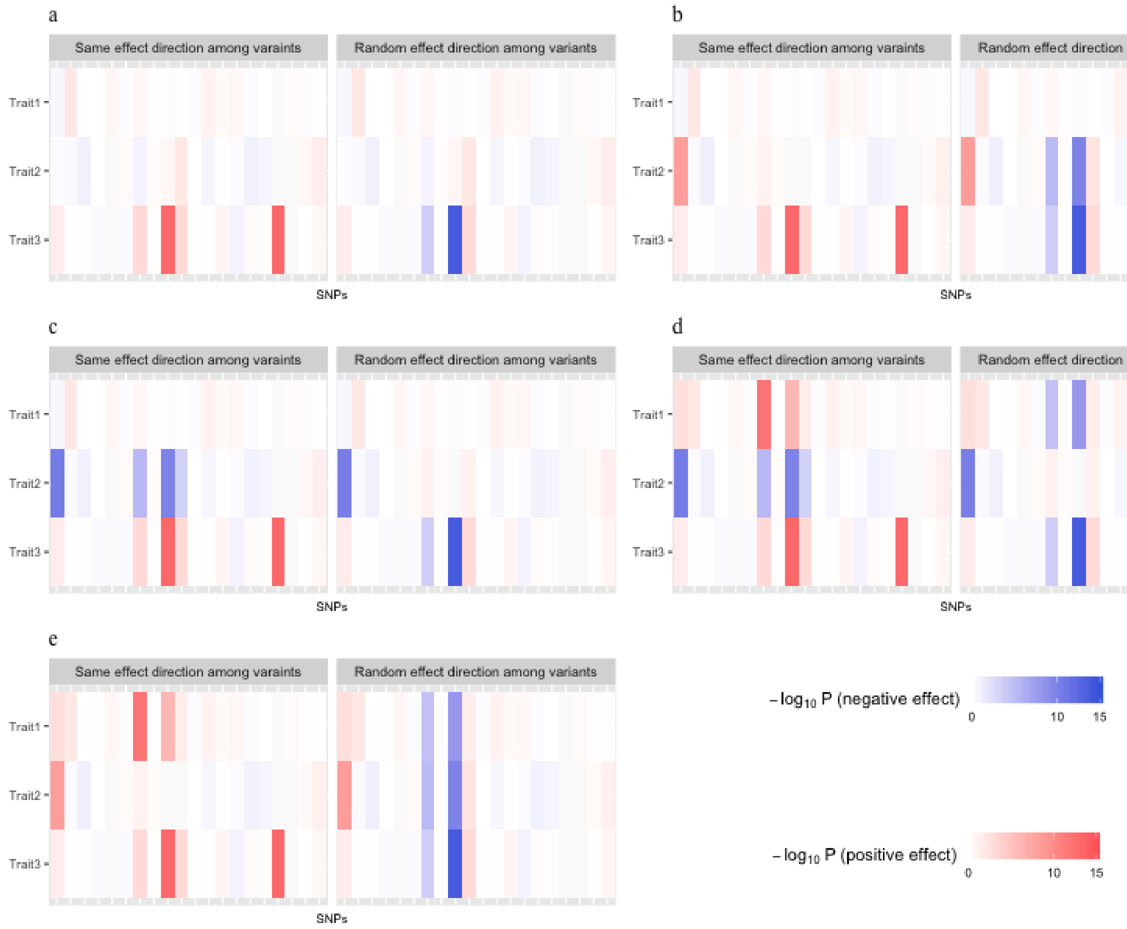

Supplementary Figure 7 Heat maps of association signals in the simulation settings. For each simulation setting, a heat map is generated based on a simulated dataset. The dataset contains 20 variants with 20% casual variants. Each subfigure indicates a pattern of among-trait effect directions: **a.** (0, 0, 1); **b.** (0, 1, 1); **c.** (0, -1, 1); **d.** (1, -1, 1); **e.** (1, 1, 1). Within each subfigure, the left column assumes the effects of the causal variant have the same direction and the right column assumes the effect directions are randomly assigned. The darkness of the color indicates the variant-level Z-test P-values (in $-\log_{10}$ scale) for individual traits. The positive and negative Z-scores are indicated by red and blue colors, respectively.

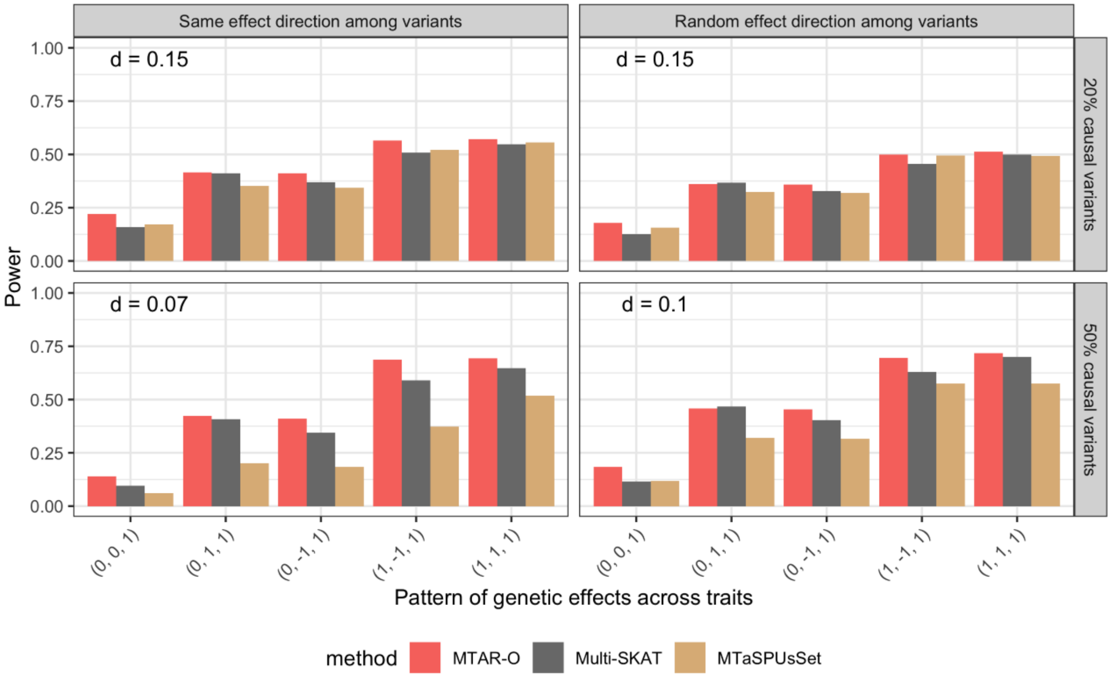

Supplementary Figure 8 Power comparisons of MTAR-O, Multi-SKAT, and MTaSPUsSet. Each bar represents the empirical power for a method estimated as the proportion of P-values less than 1.0×10^{-4} based on 10^4 replicates. The percentage of causal variants is set to be 20% or 50%, which corresponds to the two rows. The left column assumes the effects of the causal variants have the same direction, whereas the right column assumes the effect directions are randomly determined with an equal probability. The effect sizes ($|\beta_{kj}|$'s) of the causal variants have a decreasing relationship with MAF as $|\beta_{kj}| = d|\log_{10} \text{MAF}_j|$, where the constant d depends on the percentage of causal variants and the direction of their effects (the value of d is presented in each subfigure). For each configuration in a subfigure, five patterns of among-trait effects are considered.

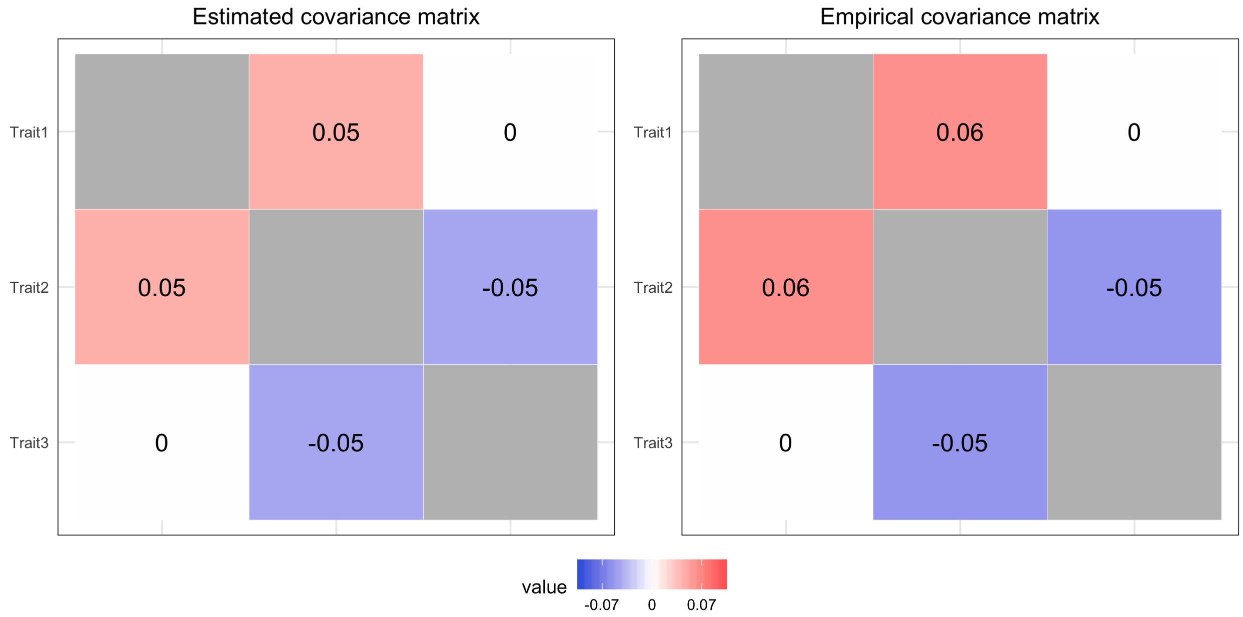

Supplementary Figure 9 Benchmarking the proposed covariance estimates of among-trait Z-score summary statistics due to sample overlap against empirical sample covariance of Z-score over 10^4 simulated datasets. The estimated covariance matrix is the average value of $\widehat{\zeta}_{kk'}$ (Methods) across 10^4 simulation replicates. The empirical covariance matrix is the sample covariance of Z-score among traits calculated from the same 10^4 replicates.

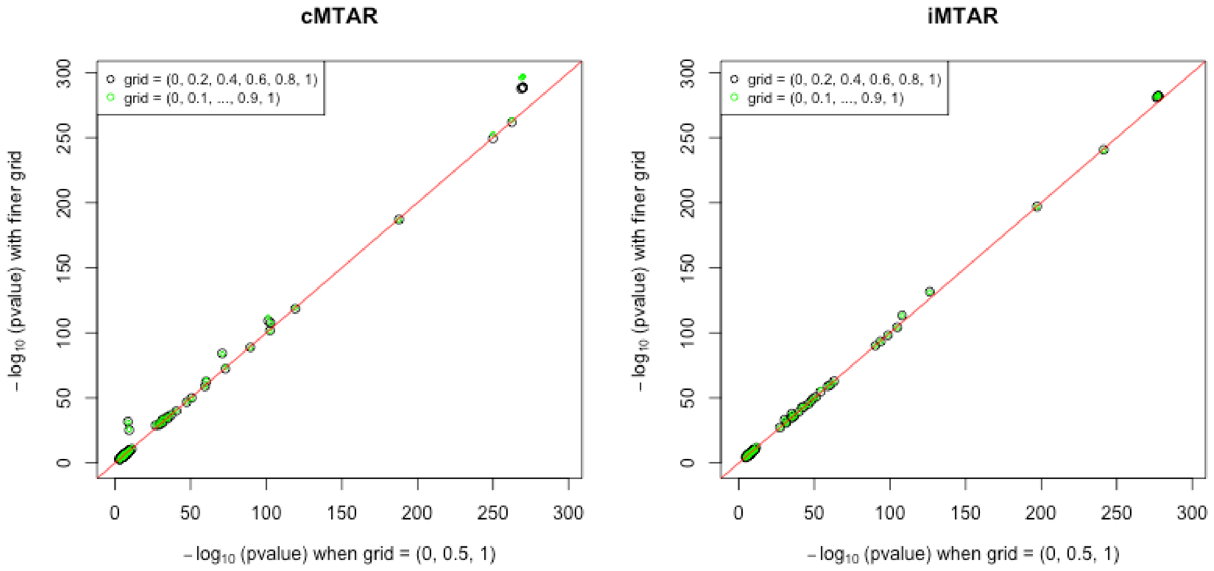

Supplementary Figure 10 Comparison of cMTAR (or iMTAR) P-values using different grid resolutions in the GLGC data analysis. Three different grid resolutions were considered: 1) $\rho_1 = \rho_2 = (0, 0.5, 1)$; 2) $\rho_1 = \rho_2 = (0, 0.2, \ldots, 0.8, 1)$; and 3) $\rho_1 = \rho_2 = (0, 0.1, \ldots, 0.9, 1)$. The left figure shows the cMTAR results and right figure shows the iMTAR results. Only the significant genes identified by these two tests are shown.

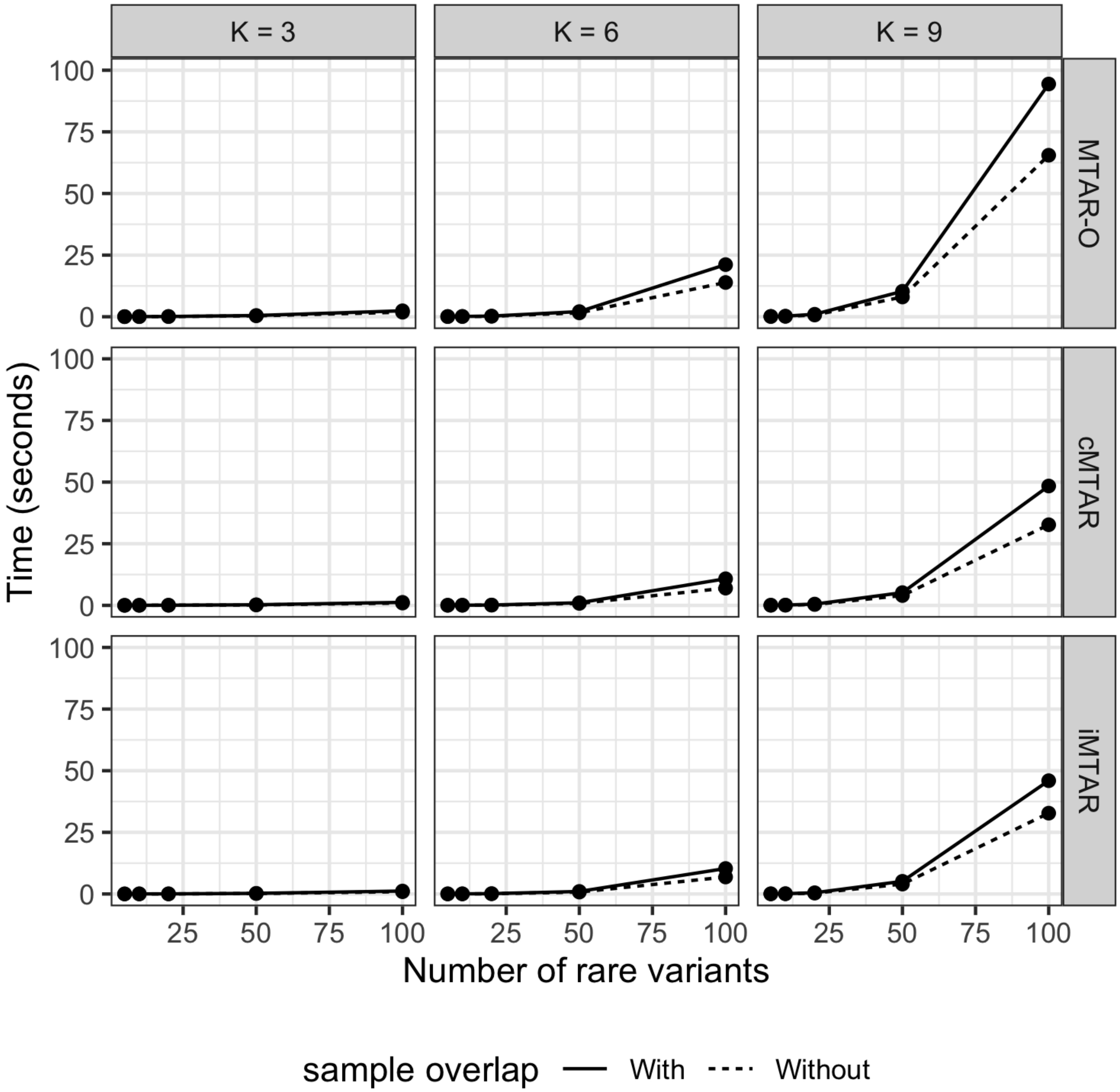

Supplementary Figure 11 Computation time of MTAR-O, cMTAR, and iMTAR as the function of the number of rare variants. We considered the analyses of 3, 6 and 9 traits and recorded the time of performing MTAR-O, cMTAR and iMTAR tests starting from the summary statistics. Solid and dashed curves represent tests with and without accounting for sample overlap.

Supplementary Tables

Supplementary Table 1 Summary of features in multi-trait multi-variant methods.

	Rare variant association analysis	Use summary statistics	Handle various patterns of sample overlap across traits	Fast p-value computation (no permutation or MC simulation required)
MTAR	✓	✓	✓	✓
MTaSPUsSet [1]		✓		
metaCCA [2]<br>MGAS [3]		✓		✓
Multi-SKAT [4]<br>DKAT [5]<br>MARV [6]<br>MAAUSS [7]<br>MSKAT [8]<br>GAMuT [9]	✓			✓

Supplementary Table 2 *P*-values of the 140 significant genes detected by at least one of the five methods MTAR-O, cMTAR, iMTAR, cctP, and minP.

Chr	Gene	Size	MTAR-O	cMTAR	iMTAR	cctP	minP
1	AMIGO1	5	1.33E-09	2.57E-06	1.71E-09	5.95E-10	5.95E-10
1	CELSR2	55	5.22E-99	1.16E-73	2.49E-99	5.79E-99	5.79E-99
1	COL24A1	43	1.57E-07	1.44E-07	8.28E-08	7.25E-05	1.41E-04
1	MCL1	6	2.33E-08	2.61E-07	8.00E-09	1.25E-05	1.49E-05
1	MYBPHL	18	7.17E-39	4.49E-10	1.95E-37	2.42E-39	2.42E-39
1	PARS2	10	4.25E-08	4.94E-05	4.31E-07	1.47E-08	1.58E-08
1	PCSK9	24	0.00E+00	2.32E-269	0.00E+00	0.00E+00	0.00E+00
1	SARS	7	2.09E-13	3.22E-08	2.74E-11	6.97E-14	6.96E-14
1	SYPL2	7	6.94E-08	3.98E-06	2.61E-08	2.12E-07	2.13E-07
2	ABCG5	18	1.35E-11	5.19E-08	5.02E-10	4.54E-12	4.54E-12
2	AGBL5	18	6.26E-10	1.08E-09	4.56E-10	6.00E-10	6.00E-10
2	APOB	122	9.93E-270	1.81E-244	3.31E-270	3.38E-178	3.38E-178
2	ASB3—GPR75-ASB3	14	4.47E-09	1.26E-04	1.49E-09	3.97E-06	3.98E-06
2	EMILIN1	24	3.29E-07	2.59E-04	1.85E-06	1.17E-07	1.17E-07
2	GCKR	22	6.66E-15	3.82E-10	3.59E-11	2.22E-15	2.36E-15
2	IFT172	42	4.15E-09	7.18E-04	5.99E-08	1.42E-09	1.42E-09
2	SNX17	5	9.06E-07	1.89E-06	6.53E-07	7.98E-07	1.27E-06
2	TSPYL6	10	6.79E-10	8.97E-10	3.03E-10	1.73E-06	1.76E-06
3	CDC25A	8	1.34E-09	1.86E-06	1.88E-09	5.89E-10	6.00E-10
3	DNAH1	106	3.22E-32	2.33E-32	2.00E-32	2.99E-07	3.94E-07
3	GRM2	14	3.43E-07	1.38E-07	9.14E-07	2.60E-06	2.60E-06
3	HEMK1	7	8.19E-08	5.46E-08	6.83E-08	2.74E-07	2.74E-07
3	ITIH3	24	2.55E-06	8.42E-06	9.46E-07	1.17E-03	1.74E-03
3	ITIH4	24	4.49E-10	1.18E-09	2.40E-10	5.98E-10	6.00E-10
3	NISCH	25	1.98E-09	2.12E-09	9.59E-10	7.46E-07	7.50E-07
3	STAB1	62	9.82E-12	1.13E-11	4.61E-12	6.32E-06	7.00E-06
4	FGB	9	4.18E-10	4.63E-10	2.99E-10	5.99E-10	5.99E-10
4	HGFAC	23	4.82E-07	1.29E-06	4.76E-07	2.99E-07	3.01E-07
4	MTTP	16	3.34E-06	1.15E-06	3.52E-05	6.63E-04	6.64E-04
4	PLA2G12A	2	8.67E-09	7.71E-09	4.63E-09	4.10E-06	5.97E-06
5	SPARC	6	3.23E-06	2.63E-06	1.84E-06	2.77E-04	2.86E-04
6	BTNL2	21	5.84E-10	7.86E-10	2.84E-10	2.95E-09	2.95E-09
6	C2	17	6.44E-10	8.77E-10	2.84E-10	2.06E-05	3.38E-05
6	C6orf10	9	1.22E-06	1.02E-06	6.82E-07	8.57E-05	8.68E-05
6	FRMD1	29	6.17E-09	4.49E-09	3.92E-09	1.24E-07	1.24E-07
6	HFE	12	4.80E-06	1.48E-03	1.94E-05	1.75E-06	1.75E-06
6	HLA-DQB1	5	5.12E-08	4.61E-08	2.71E-08	3.67E-04	4.51E-04
6	LPA	35	1.18E-37	1.59E-29	9.56E-36	3.93E-38	3.93E-38
6	MCCD1	3	2.30E-07	2.55E-07	1.18E-07	1.55E-06	1.55E-06
6	MICA	9	7.01E-10	7.65E-10	3.60E-10	5.32E-09	5.32E-09
6	MSH5	14	5.78E-07	4.85E-06	2.20E-07	2.27E-06	2.28E-06
6	NFKBIL1	3	9.40E-36	1.48E-35	3.98E-36	2.22E-15	2.04E-15
6	NOTCH4	37	1.47E-08	5.27E-04	4.91E-09	6.28E-05	8.10E-05

Chr	Gene	Size	MTAR-O	cMTAR	iMTAR	cctP	minP
6	PRRC2A	39	3.64E-48	4.55E-48	1.66E-48	2.99E-10	6.00E-10
6	SKIV2L	20	7.71E-10	7.61E-06	4.50E-10	6.00E-10	6.00E-10
6	SLC22A1	27	2.59E-11	6.34E-09	1.52E-10	9.16E-12	9.30E-12
6	SPACA1	4	8.06E-61	5.89E-61	4.94E-61	1.30E-40	1.30E-40
6	TNXB	57	2.20E-51	2.31E-51	1.08E-51	2.21E-31	2.21E-31
6	ZNF76	22	7.71E-07	2.61E-07	1.65E-05	2.10E-03	2.73E-03
7	CD36	26	5.10E-42	5.15E-38	1.74E-42	8.05E-41	8.05E-41
7	KIAA1324L	12	2.10E-08	1.82E-08	1.14E-08	3.03E-05	3.24E-05
7	NPC1L1	37	1.80E-09	1.02E-03	9.28E-07	6.00E-10	6.00E-10
8	LPL	12	0.00E+00	0.00E+00	0.00E+00	7.93E-244	7.93E-244
8	ZNF572	15	1.26E-06	1.82E-06	5.49E-07	1.09E-04	1.61E-04
9	ABCA1	37	1.78E-41	3.51E-09	1.13E-40	6.25E-42	6.25E-42
10	A1CF	4	5.33E-10	8.79E-10	2.97E-10	8.83E-10	8.83E-10
10	ASCC1	12	8.09E-10	1.27E-09	3.44E-10	7.33E-08	8.64E-08
11	APOA1	4	2.35E-30	1.34E-27	7.89E-31	1.40E-28	1.45E-28
11	APOA4	15	3.09E-63	4.01E-60	1.03E-63	2.26E-53	2.26E-53
11	APOA5	9	3.26E-108	1.68E-103	1.09E-108	9.10E-79	9.10E-79
11	APOC3	4	0.00E+00	0.00E+00	0.00E+00	8.80E-245	8.80E-245
11	BUD13	15	4.32E-10	8.85E-10	2.22E-10	7.61E-10	7.61E-10
11	CKAP5	16	4.90E-06	3.97E-06	2.79E-06	6.53E-04	6.68E-04
11	CREB3L1	9	1.85E-07	1.62E-07	1.01E-07	6.08E-06	6.14E-06
11	DSCAML1	25	1.01E-06	1.53E-06	4.41E-07	2.53E-05	2.53E-05
11	KBTBD4	4	2.47E-08	3.97E-08	1.08E-08	2.63E-07	2.70E-07
11	MEN1	4	4.14E-06	3.91E-06	2.23E-06	4.97E-05	6.98E-05
11	NR1H3	7	3.59E-07	3.64E-07	1.79E-07	3.26E-05	3.27E-05
11	OR8J1	6	1.25E-08	1.30E-08	6.18E-09	1.84E-06	1.85E-06
11	OR8U1—OR8U8	7	3.17E-06	3.12E-06	1.60E-06	6.11E-04	1.19E-03
11	PAFAH1B2	3	5.13E-105	3.58E-103	1.72E-105	5.47E-54	5.47E-54
11	PCSK7	12	5.75E-10	1.52E-09	3.48E-10	5.94E-10	5.94E-10
11	PLCB3	14	2.72E-09	2.46E-09	1.44E-09	5.08E-05	7.96E-05
11	PNPLA2	14	5.23E-08	5.37E-08	2.60E-08	3.33E-06	3.34E-06
11	SIDT2	21	5.07E-06	7.31E-06	3.05E-06	7.92E-06	8.20E-06
11	TIRAP	12	4.12E-10	7.57E-10	1.73E-10	5.85E-09	5.89E-09
11	TNKS1BP1	43	1.33E-06	1.04E-02	1.04E-06	7.75E-07	7.75E-07
11	TSGA10IP	15	7.10E-09	7.78E-09	3.40E-09	1.99E-04	2.76E-04
11	ZNF259	10	2.37E-07	1.66E-06	1.22E-07	2.59E-07	3.22E-07
12	ACADS	8	4.30E-06	1.65E-05	2.45E-06	4.38E-06	5.80E-06
12	ACVRL1	8	5.13E-06	4.50E-06	2.77E-06	6.12E-04	9.73E-04
12	C12orf41	4	1.16E-06	1.00E-06	6.37E-07	8.19E-05	1.04E-04
12	CCDC62	11	8.25E-10	5.72E-06	4.50E-10	7.08E-10	7.08E-10
12	CMAS	3	7.83E-07	6.79E-07	4.29E-07	3.62E-05	3.65E-05
12	HIP1R	28	2.11E-07	4.27E-07	1.83E-07	1.55E-07	1.55E-07
12	MPHOSPH9	23	5.47E-08	8.45E-06	1.23E-06	1.85E-08	1.85E-08
12	PRKAG1	4	4.67E-09	4.50E-09	2.38E-09	1.30E-06	1.88E-06
12	RAB21	3	1.03E-06	4.60E-04	9.19E-06	3.57E-07	6.90E-07
12	SCARB1	13	3.15E-23	3.84E-10	2.44E-11	1.05E-23	1.05E-23

Chr	Gene	Size	MTAR-O	cMTAR	iMTAR	cctP	minP
12	SH2B3	15	2.25E-06	2.48E-06	1.10E-06	4.63E-05	4.67E-05
13	SLC25A30	6	1.66E-09	6.86E-06	1.03E-08	5.86E-10	6.00E-10
14	DDHD1	9	6.51E-07	5.74E-07	3.50E-07	1.48E-04	1.85E-04
14	PCK2	34	2.47E-06	3.16E-06	1.11E-06	4.00E-04	5.91E-04
14	SERPINA1	14	2.08E-10	3.97E-10	1.07E-10	3.89E-10	5.99E-10
15	ARRDC4	8	5.21E-06	5.26E-06	2.60E-06	6.84E-04	1.97E-03
15	LIPC	18	3.64E-59	2.30E-33	1.21E-59	6.79E-48	6.79E-48
15	MAP1A	48	1.38E-90	3.08E-90	5.40E-91	1.71E-52	1.71E-52
15	RNF111	16	1.09E-31	2.23E-10	5.44E-32	1.09E-31	1.09E-31
16	ANKS3	20	5.79E-10	9.00E-10	3.00E-10	1.35E-09	1.35E-09
16	CES3	21	4.90E-10	8.93E-10	3.00E-10	5.99E-10	5.99E-10
16	CETP	12	0.00E+00	0.00E+00	0.00E+00	0.00E+00	0.00E+00
16	CFDP1	7	1.58E-06	1.40E-06	8.47E-07	9.08E-04	1.11E-03
16	DHX38	16	1.47E-06	5.33E-04	7.83E-06	5.24E-07	5.24E-07
16	DPEP3	8	4.18E-09	1.73E-08	2.98E-09	3.09E-09	3.09E-09
16	ELMO3	18	4.79E-10	7.90E-10	3.00E-10	6.00E-10	6.00E-10
16	FAM65A	15	6.80E-35	9.45E-32	2.27E-35	1.24E-32	1.25E-32
16	LCAT	2	2.64E-28	1.55E-10	5.35E-28	1.05E-28	1.05E-28
16	NLRC5	38	2.11E-38	8.65E-31	1.43E-35	7.05E-39	7.05E-39
16	PLEKHG4	28	3.80E-10	8.50E-10	2.00E-10	5.80E-10	5.99E-10
16	PMFBP1	24	8.73E-07	2.95E-03	5.29E-04	2.91E-07	2.92E-07
16	SLC12A3	32	9.37E-51	3.19E-41	1.20E-49	3.21E-51	3.21E-51
16	TMED6	10	1.15E-07	1.70E-06	1.25E-07	5.74E-08	5.74E-08
16	ZNF500	17	3.26E-09	7.07E-09	1.28E-09	2.96E-06	3.34E-06
17	ABCA6	42	5.28E-55	2.41E-30	9.95E-55	2.14E-55	2.14E-55
17	APOH	10	2.93E-46	8.66E-10	9.77E-47	2.31E-40	2.31E-40
17	BECN1	4	2.43E-06	5.29E-06	9.55E-07	1.05E-03	2.37E-03
17	CD300LG	9	3.61E-197	3.03E-188	1.20E-197	5.17E-114	5.17E-114
17	G6PC	8	1.20E-13	5.00E-10	2.37E-11	4.00E-14	3.99E-14
17	GEMIN4	35	1.52E-06	7.23E-04	5.09E-07	3.61E-04	6.14E-04
17	SHBG	7	2.53E-06	3.15E-06	1.49E-06	5.07E-06	6.26E-06
18	LIPG	11	1.70E-126	6.27E-102	5.65E-127	2.13E-109	2.13E-109
19	ANGPTL4	9	0.00E+00	0.00E+00	0.00E+00	1.07E-206	1.07E-206
19	APOC2	6	2.55E-06	1.70E-04	3.46E-05	8.77E-07	8.77E-07
19	APOE	3	3.56E-43	1.35E-35	1.19E-43	3.13E-18	3.13E-18
19	AXL	11	1.24E-06	1.01E-06	7.01E-07	1.78E-04	1.82E-04
19	BCAM	29	0.00E+00	3.76E-263	0.00E+00	0.00E+00	0.00E+00
19	CBLC	7	1.66E-241	7.16E-120	5.54E-242	7.84E-228	7.84E-228
19	DOCK6	39	2.89E-06	8.33E-02	3.34E-05	9.93E-07	9.93E-07
19	KRI1	22	7.71E-10	2.98E-04	4.50E-10	6.00E-10	6.00E-10
19	LAIR1	13	2.50E-07	2.09E-07	1.39E-07	7.56E-05	9.51E-05
19	LDLR	24	1.58E-06	4.34E-04	3.06E-05	5.36E-07	5.49E-07
19	LOC55908	7	8.23E-10	9.00E-10	3.94E-10	9.45E-06	1.46E-05
19	POLD1	24	3.61E-10	3.00E-10	3.00E-10	6.09E-10	6.09E-10
19	ZGLP1	6	2.66E-07	1.34E-06	2.79E-07	1.44E-07	1.44E-07
19	ZNF222	10	5.56E-08	4.31E-04	7.83E-07	1.90E-08	1.90E-08

Chr	Gene	Size	MTAR-O	cMTAR	iMTAR	cctP	minP
20	HNF4A	7	5.82E-94	1.37E-71	1.94E-94	2.99E-81	2.99E-81
20	PCK1	25	5.70E-07	7.13E-06	1.60E-06	2.22E-07	2.22E-07
21	COL18A1	44	5.85E-08	4.36E-08	3.52E-08	6.97E-05	7.06E-05
22	FBLN1	18	1.29E-07	1.13E-03	2.91E-06	4.38E-08	4.38E-08
22	PPARA	6	6.22E-10	7.00E-10	3.22E-10	3.41E-09	3.41E-09

Supplementary Table 3 Replication association results using the UK Biobank Neale v2 summary statistics.

Chr	Gene[1]	Size[2]	MTAR-O	cMTAR	iMTAR	cctP	minP	SKAT.LDL	SKAT.HDL	SKAT.TG	Burden.LDL	Burden.HDL	Burden.TG
1	COL24A1	23	5.13E-01	6.15E-01	5.45E-01	3.80E-01	1.23E+00	5.73E-01	4.44E-01	4.38E-01	2.06E-01	4.58E-01	2.89E-01
2	ASB3—GPR75-ASB3	5	1.83E-03	4.93E-03	3.93E-03	8.49E-04	8.73E-04	5.39E-01	1.45E-04	5.55E-03	4.42E-01	8.08E-02	3.08E-01
5	SPARC	5	1.70E-03	1.97E-03	1.09E-03	2.91E-03	4.60E-03	4.71E-01	7.66E-04	2.71E-01	5.17E-01	1.33E-03	1.99E-01
6	C2	7	1.43E-03	4.46E-01	2.12E-01	4.79E-04	7.85E-04	1.31E-04	1.09E-02	9.15E-04	1.52E-02	5.83E-02	2.76E-04
11	OR8U1—OR8U8	4	1.51E-11	4.31E-10	1.49E-10	5.28E-12	5.33E-12	1.35E-01	9.97E-11	5.93E-05	4.33E-01	8.88E-13	4.71E-04
11	PNPLA2	5	6.33E-08	1.17E-02	4.36E-02	2.11E-08	2.11E-08	3.64E-02	3.52E-09	1.31E-04	4.24E-02	8.16E-02	4.08E-01
12	ACVRL1	5	2.44E-02	3.93E-02	3.65E-02	1.42E-02	3.27E-02	4.27E-02	7.86E-03	1.73E-02	4.75E-02	5.44E-03	1.08E-01
12	C12orf41	2	3.11E-02	2.19E-02	9.29E-02	2.50E-02	3.72E-02	2.35E-02	2.43E-01	1.26E-01	6.20E-03	3.53E-01	4.19E-02
15	ARRDC4	4	1.21E-04	1.37E-04	7.05E-05	3.00E-04	3.98E-04	6.77E-01	6.63E-05	2.19E-01	8.45E-01	2.02E-04	6.99E-01
16	CFDP1	4	2.22E-02	3.17E-01	4.17E-01	7.56E-03	9.32E-03	1.28E-01	6.57E-01	6.87E-03	4.87E-01	6.19E-01	1.55E-03
19	AXL	3	2.79E-05	4.96E-02	6.55E-02	9.30E-06	1.01E-05	1.55E-03	1.28E-01	1.69E-06	5.90E-04	2.46E-01	1.98E-05

1. The genes BECN1, CAMS and DDHD1 with cumulative minor allele count less than 10 in the UK Biobank are not analyzed.
2. We used all the rare SNPs with MAF less than 5% in our replication analysis. The result from a more conservative replication is very similar, where only rare SNPs presenting in GLGC are analyzed.

Supplementary Table 4 Empirical type I error rates.

α	MTAR-O	cMTAR	iMTAR	cctP	minP
1.00E-04	1.07E-04	1.03E-04	1.09E-04	9.50E-05	9.80E-05
1.00E-05	1.04E-05	1.01E-05	1.07E-05	9.67E-06	9.90E-06
2.50E-06	2.46E-06	2.45E-06	2.74E-06	2.32E-06	2.37E-06

Each cell represents the empirical type I error rate for a method estimated as the proportion of P-values less than α under the null hypothesis based on 1E8 replicates of simulation.

Supplementary Table 5 Association evidence for the traits LDL, HDL and TG in the previous GLGC data analysis [10].

POSITION	RS	REF/ALT	Gene	Annotation	N	ALT.FREQ	Trait	Z-test P.value	Beta	SE	I2.Q.P.value.for.Q
11:64031241	rs35169799	C/T	PLCB3	Ser778Leu	314415	0.06	HDL	4.00E-13	-0.039	0.0054	2%,95.94,1
11:64031241	rs35169799	C/T	PLCB3	Ser778Leu	293853	0.059	LDL	0.3	0.0058	0.0056	0%,77.41,0.52
11:64031241	rs35169799	C/T	PLCB3	Ser778Leu	303685	0.059	TG	3.10E-12	0.038	0.0055	2.1%,93.97,0.69
3:52532118	rs13326165	A/G	STAB1	Intron	316391	0.81	HDL	3.00E-14	-0.025	0.0032	0%,94.77,0.6
3:52532118	rs13326165	A/G	STAB1	Intron	295826	0.81	LDL	0.33	0.0033	0.0034	0%,91.41,0.5
3:52532118	rs13326165	A/G	STAB1	Intron	305699	0.81	TG	9.70E-10	0.02	0.0033	3.5%,98.49,0.78
11:47290147	rs61731956	G/A	NR1H3	Arg370Gln	308466	0.00029	HDL	5.10E-10	0.47	0.076	11.3%,56.37,0.021
11:47290147	rs61731956	G/A	NR1H3	Arg370Gln	287937	0.00028	LDL	0.041	-0.16	0.08	0%,40.64,0.42
11:47290147	rs61731956	G/A	NR1H3	Arg370Gln	297736	0.00028	TG	0.00011	-0.31	0.079	20.4%,59.05,0.052
12:111884608	rs3184504	T/C	SH2B3	Trp262Arg	295475	0.57	HDL	1.10E-21	0.027	0.0028	0%,73.15,0.95
12:111884608	rs3184504	T/C	SH2B3	Trp262Arg	276356	0.57	LDL	5.80E-21	0.027	0.0029	1.6%,93.52,0.5
12:111884608	rs3184504	T/C	SH2B3	Trp262Arg	284767	0.57	TG	0.00011	-0.011	0.0029	0%,78.85,0.77
19:11350874	rs145464906	C/T	LOC55908	Gln121Stp	308067	0.00068	HDL	5.00E-18	0.43	0.05	4%,55.23,0.41
19:11350874	rs145464906	C/T	LOC55908	Gln121Stp	287558	0.00069	LDL	0.25	-0.059	0.051	4.8%,53.58,0.085
19:11350874	rs145464906	C/T	LOC55908	Gln121Stp	297329	0.00068	TG	2.80E-12	-0.35	0.051	0%,50.95,0.31
4:100504664	rs3816873	T/C	MTTP	Ile128Thr	316391	0.26	HDL	0.0031	-0.0086	0.0029	9.7%,107.46,0.18
4:100504664	rs3816873	T/C	MTTP	Ile128Thr	295826	0.26	LDL	1.00E-08	-0.017	0.003	4.2%,99.2,0.98
4:100504664	rs3816873	T/C	MTTP	Ile128Thr	305699	0.26	TG	0.25	-0.0034	0.003	1.7%,96.69,0.55
11:117299414	rs145244816	C/T	DSCAML1	Arg1991Gln	301025	0.00024	HDL	3.00E-08	0.45	0.082	17%,39.75,0.023
11:117299414	rs145244816	C/T	DSCAML1	Arg1991Gln	280551	0.00025	LDL	0.18	0.11	0.083	0%,22.93,0.85
11:117299414	rs145244816	C/T	DSCAML1	Arg1991Gln	290277	0.00025	TG	1.20E-13	-0.61	0.082	14.8%,37.57,0.0016
21:46875775	rs114139997	G/A	COL18A1	Gly111Arg	295703	0.0011	HDL	0.00027	0.14	0.039	31.4%,27.71,0.32
21:46875775	rs114139997	G/A	COL18A1	Gly111Arg	275255	0.0012	LDL	0.043	-0.08	0.039	26.5%,25.85,0.32
21:46875775	rs114139997	G/A	COL18A1	Gly111Arg	284962	0.0012	TG	7.90E-21	-0.37	0.039	27.3%,26.14,0.01
21:46875817	rs200559406	G/A	COL18A1	Val125Ile	308466	0.00087	HDL	4.40E-07	-0.22	0.043	0%,55.7,0.74
21:46875817	rs200559406	G/A	COL18A1	Val125Ile	287937	0.00089	LDL	0.1	0.072	0.044	0%,59.51,0.38
21:46875817	rs200559406	G/A	COL18A1	Val125Ile	297736	9.00E-04	TG	6.00E-12	0.3	0.043	13.6%,71.78,0.67
6:32261252	rs7775397	T/G	C6orf10	Glu317Ala	313891	0.086	HDL	0.00064	-0.017	0.0049	0%,86.57,0.39
6:32261252	rs7775397	T/G	C6orf10	Glu317Ala	293327	0.085	LDL	0.00096	-0.017	0.0051	0%,87.54,0.075
6:32261252	rs7775397	T/G	C6orf10	Glu317Ala	303101	0.085	TG	6.30E-13	-0.036	0.005	16.3%,107.53,0.13

Supplementary References

1. Kwak, I.-Y. & Pan, W. Gene-and pathway-based association tests for multiple traits with GWAS summary statistics. *Bioinformatics* **33**, 64–71 (2016).

2. Cichonska, A. et al. metaCCA: summary statistics-based multivariate meta-analysis of genome-wide association studies using canonical correlation analysis. *Bioinformatics* **32**, 1981–1989 (2016).

3. Van der Sluis, S. et al. MGAS: a powerful tool for multivariate gene-based genome-wide association analysis. *Bioinformatics* **31**, 1007–1015 (2014).

4. Dutta, D., Scott, L., Boehnke, M. & Lee, S. Multi-SKAT: General framework to test for rare-variant association with multiple phenotypes. *Genet. Epidemiol.* **43**, 4–23 (2019).

5. Zhan, X. et al. Powerful genetic association analysis for common or rare variants with high-dimensional structured traits. *Genetics* **206**, 1779–1790 (2017).

6. Kaakinen, M. et al. MARV: a tool for genome-wide multi-phenotype analysis of rare variants. *BMC Bioinform.* **18**, 110 (2017).

7. Lee, S. et al. Rare variant association test with multiple phenotypes. *Genet. Epidemiol.* **41**, 198–209 (2017).

8. Wu, B. & Pankow, J. S. Sequence kernel association test of multiple continuous

phenotypes. *Genet. Epidemiol.* **40**, 91–100 (2016).

9. Broadaway, K. A. et al. A statistical approach for testing cross-phenotype effects of rare variants. *Am. J. Hum. Genet.* **98**, 525–540 (2016).

10. Liu, D. J. et al. Exome-wide association study of plasma lipids in $>$ 300,000 individuals. *Nat. Genet.* **49**, 1758–1766 (2017).
