# Supplementary material for: Multi-trait analysis of rare-variant association summary statistics using MTAR
Source: Nat Commun. 2020 Jun 5;11:2850. doi: 10.1038/s41467-020-16591-0 (PMC7275056; doi:10.1038/s41467-020-16591-0)
Supplement: Supplementary file 3 — Description of Additional Supplementary Files [file 41467_2020_16591_MOESM3_ESM.docx]

**Description of Additional Supplementary Files**

**File name:** Supplementary Data 1

**Description:** Gene set enrichment analysis results

**File name:** Supplementary Data 2

**Description:** Association evidence from the Open Targets database for the traits LDL, HDL and TG

**File name:** Supplementary Data 3

**Description:** Association evidence from the STOPGAP database for the traits LDL, HDL and TG
