# Supplementary material for: Multi-trait analysis of rare-variant association summary statistics using MTAR
Source: Nat Commun. 2020 Jun 5;11:2850. doi: 10.1038/s41467-020-16591-0 (PMC7275056; doi:10.1038/s41467-020-16591-0)
Supplement: Supplementary file 7 — Reporting Summary [file 41467_2020_16591_MOESM7_ESM.pdf]

nature research

Corresponding author(s): Zheng-Zheng Tang

Last updated by author(s): May 1, 2020

# Reporting Summary

Nature Research wishes to improve the reproducibility of the work that we publish. This form provides structure for consistency and transparency in reporting. For further information on Nature Research policies, see Authors & Referees and the Editorial Policy Checklist .

## Statistics

For all statistical analyses, confirm that the following items are present in the figure legend, table legend, main text, or Methods section.

| n/a | Confirmed | |
|---|---|---|
| ☐ | ☒ | The exact sample size (*n*) for each experimental group/condition, given as a discrete number and unit of measurement |
| ☐ | ☒ | A statement on whether measurements were taken from distinct samples or whether the same sample was measured repeatedly |
| ☐ | ☒ | The statistical test(s) used AND whether they are one- or two-sided<br>*Only common tests should be described solely by name; describe more complex techniques in the Methods section.* |
| ☐ | ☒ | A description of all covariates tested |
| ☐ | ☒ | A description of any assumptions or corrections, such as tests of normality and adjustment for multiple comparisons |
| ☐ | ☒ | A full description of the statistical parameters including central tendency (e.g. means) or other basic estimates (e.g. regression coefficient) AND variation (e.g. standard deviation) or associated estimates of uncertainty (e.g. confidence intervals) |
| ☐ | ☒ | For null hypothesis testing, the test statistic (e.g. *F*, *t*, *r*) with confidence intervals, effect sizes, degrees of freedom and *P* value noted<br>*Give P values as exact values whenever suitable.* |
| ☒ | ☐ | For Bayesian analysis, information on the choice of priors and Markov chain Monte Carlo settings |
| ☒ | ☐ | For hierarchical and complex designs, identification of the appropriate level for tests and full reporting of outcomes |
| ☒ | ☐ | Estimates of effect sizes (e.g. Cohen's *d*, Pearson's *r*), indicating how they were calculated |

*Our web collection on statistics for biologists contains articles on many of the points above.*

## Software and code

Policy information about availability of computer code

| | |
|---|---|
| Data collection | The main focus of this paper is to propose a statistical method, with applications to existing data. Therefore no software was used to collect the data |
| Data analysis | The MTAR R package is available at the Comprehensive R Archive Network (CRAN): https://cran.r-project.org/web/packages/MTAR<br>Other software programs used in the data analysis are: SKAT R package v1.3.2.1; MultiSKAT R package v1.0; aSPU R package v1.48; FUMA v1.3.5; TissueEnrich v1.8.0 |

For manuscripts utilizing custom algorithms or software that are central to the research but not yet described in published literature, software must be made available to editors/reviewers. We strongly encourage code deposition in a community repository (e.g. GitHub). See the Nature Research guidelines for submitting code & software for further information.

## Data

Policy information about availability of data

All manuscripts must include a data availability statement. This statement should provide the following information, where applicable:

- Accession codes, unique identifiers, or web links for publicly available datasets
- A list of figures that have associated raw data
- A description of any restrictions on data availability

The summary statistics used in case study were from Global Lipids Genetics Consortium, directly downloaded from http://csg.sph.umich.edu/abecasis/public/lipids2017/; UK Biobank GWAS summary statistics data (Neale v2) used for replication were downloaded from http://www.nealelab.is/uk-biobank (In particular, the association summary statistics for the trait LDL direct (mmol/L) were downloaded from https://www.dropbox.com/s/2msvdv4axfz362b/30780_raw.gwas.imputed_v3.both_sexes.tsv.bgz?dl=0; for the trait HDL direct (mmol/L) were downloaded from https://www.dropbox.com/s/sn30890f64p0htu/30760_raw.gwas.imputed_v3.both_sexes.tsv.bgz?dl=0; for the trait Triglycerides (mmol/L) were downloaded from, https://www.dropbox.com/s/0tdxu9g7itbct6m/30870_raw.gwas.imputed_v3.both_sexes.tsv.bgz?dl=0)

# Field-specific reporting

Please select the one below that is the best fit for your research. If you are not sure, read the appropriate sections before making your selection.

[x] Life sciences [ ] Behavioural & social sciences [ ] Ecological, evolutionary & environmental sciences

For a reference copy of the document with all sections, see nature.com/documents/nr-reporting-summary-flat.pdf

# Life sciences study design

All studies must disclose on these points even when the disclosure is negative.

| | |
|---|---|
| Sample size | This is a methodology-based research. We developed a framework for joint analysis of summary statistics from rare-variant association studies of multiple different traits. In order to discover new genes and replicate the results in the case study, we used the largest datasets available. |
| Data exclusions | No data were excluded. |
| Replication | Not applicable. |
| Randomization | Not applicable. |
| Blinding | Not applicable. |

# Reporting for specific materials, systems and methods

We require information from authors about some types of materials, experimental systems and methods used in many studies. Here, indicate whether each material, system or method listed is relevant to your study. If you are not sure if a list item applies to your research, read the appropriate section before selecting a response.

## Materials & experimental systems

| n/a | Involved in the study | |
|---|---|---|
| [x] | [ ] | Antibodies |
| [x] | [ ] | Eukaryotic cell lines |
| [x] | [ ] | Palaeontology |
| [x] | [ ] | Animals and other organisms |
| [x] | [ ] | Human research participants |
| [x] | [ ] | Clinical data |

## Methods

| n/a | Involved in the study | |
|---|---|---|
| [x] | [ ] | ChIP-seq |
| [x] | [ ] | Flow cytometry |
| [x] | [ ] | MRI-based neuroimaging |
